# Split-alignment of genomes finds orthologies more accurately

**DOI:** 10.1186/s13059-015-0670-9

**Published:** 2015-05-21

**Authors:** Martin C Frith, Risa Kawaguchi

**Affiliations:** Computational Biology Research Center (CBRC), National Institute of Advanced Industrial Science and Technology (AIST), 2-4-7 Aomi, Koto-ku, Tokyo, 135-0064 Japan; Department of Computational Biology, Faculty of Frontier Sciences, The University of Tokyo, 5-1-5 Kashiwanoha, Kashiwa, Chiba, 277-8561 Japan

## Abstract

We present a new pair-wise genome alignment method, based on a simple concept of finding an optimal *set* of local alignments. It gains accuracy by not masking repeats, and by using a statistical model to quantify the (un)ambiguity of each alignment part. Compared to previous animal genome alignments, it aligns thousands of locations differently and with much higher similarity, strongly suggesting that the previous alignments are non-orthologous. The previous methods suffer from an overly-strong assumption of long un-rearranged blocks. The new alignments should help find interesting and unusual features, such as fast-evolving elements and micro-rearrangements, which are confounded by alignment errors.

## Background

### Aim of genome alignment

**Fig. 1:**
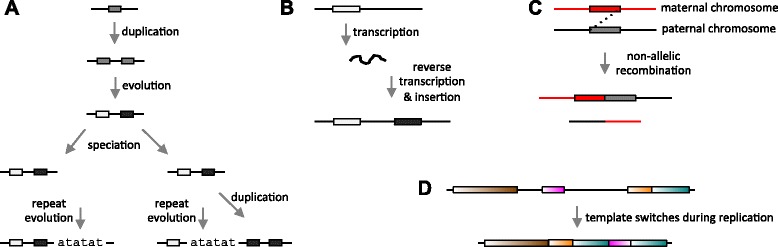
Illustrations of genome evolution. **a** Sketch of a genomic segment evolving over time, illustrating orthology and paralogy. **b** An example of asymmetric duplication (retrotransposition). **c** An example of symmetric duplication (non-allelic recombination). **d** A complex rearrangement, ascribed to three template switches during one DNA replication event [47]. The pink segment is inverted, and the green segment is duplicated

If we compare two genome sequences, such as those of human and chimp, to see how they differ, then intuitively we wish to align the “equivalent” regions of the genomes. More precisely, we wish to align orthologs, which are descended from the same sequence in the last common ancestor of the genomes. The white boxes in Fig. 1a illustrate orthologs.

We can recognize orthologs by sequence similarity, but we need to distinguish them from two other types of similar sequence. The first is paralogs, which are descended from a common ancestral sequence by intra-genome duplication *before* the speciation event. The black and white boxes in Fig. 1a are paralogous to each other. The second is independently-evolved simple sequences such as atatatatatat. Simple sequences are typically suppressed by identifying and masking them, though not all identification [1] and masking [2] procedures work equally well.

Genome comparison would be simpler if the equivalencies were always one-to-one, but unfortunately orthology is not always one-to-one. If orthologs are duplicated after the speciation event, it can be many-to-many. In Fig. 1a, the black box in the left genome is orthologous to both black boxes in the right genome.

There is a large body of ongoing research on discriminating orthologous from paralogous proteins [3–6]. A simple approach, which ignores many-to-many orthology, is to find reciprocal best matches between two proteomes. A better approach in theory (not necessarily in practice [4]) is to infer phylogenetic trees of the proteins, and thence infer speciation and duplication events. These methods are not easily adapted to whole genomes, because we must consider rearrangements causing different genomic segments to have different evolutionary relationships, and the segment boundaries are not known in advance.

### Beyond orthology?

There is a widespread desire to refine the concept of orthology, perhaps in order to avoid many-to-many equivalencies, and so people speak of “main ortholog”, “positional ortholog”, “syntenic regions”, etc [7]. These terms tend to be ill-defined. For example, “positional orthology” refers to orthologs that are in equivalent positions in two genomes: this is problematic, because the only way to define equivalent positions is by orthology. The intuition seems to be that more-extensive orthology defines equivalent positions, whereas smaller orthologous fragments do not. It is unclear how extensive the orthology has to be, or whether there is really a coherent concept here.

This has been made more precise under the term “toporthology” [7] (or “topoorthology” [8]), which is based on symmetry of duplications. For example, retrotransposition is an asymmetric duplication (Fig. 1b), because we can distinguish the original (white box) from the copy (black box). The original is the toportholog. It is important to realize that duplications can also be symmetric (Fig. 1c), so that neither duplicate is less “original” than the other: thus toporthology is not always one-to-one.

It was suggested that symmetric duplications are those where deletion of either duplicate would restore the genome to its original state [7]. However, there are cases where deletion of neither duplicate would restore the original genome (Fig. 1d). In this example, we might be tempted to say that the duplicate with longer orthologous flanking sequence is the “main ortholog”, but that simply highlights the fuzziness of the concept.

### Synteny, order and orientation

**Fig. 2:**
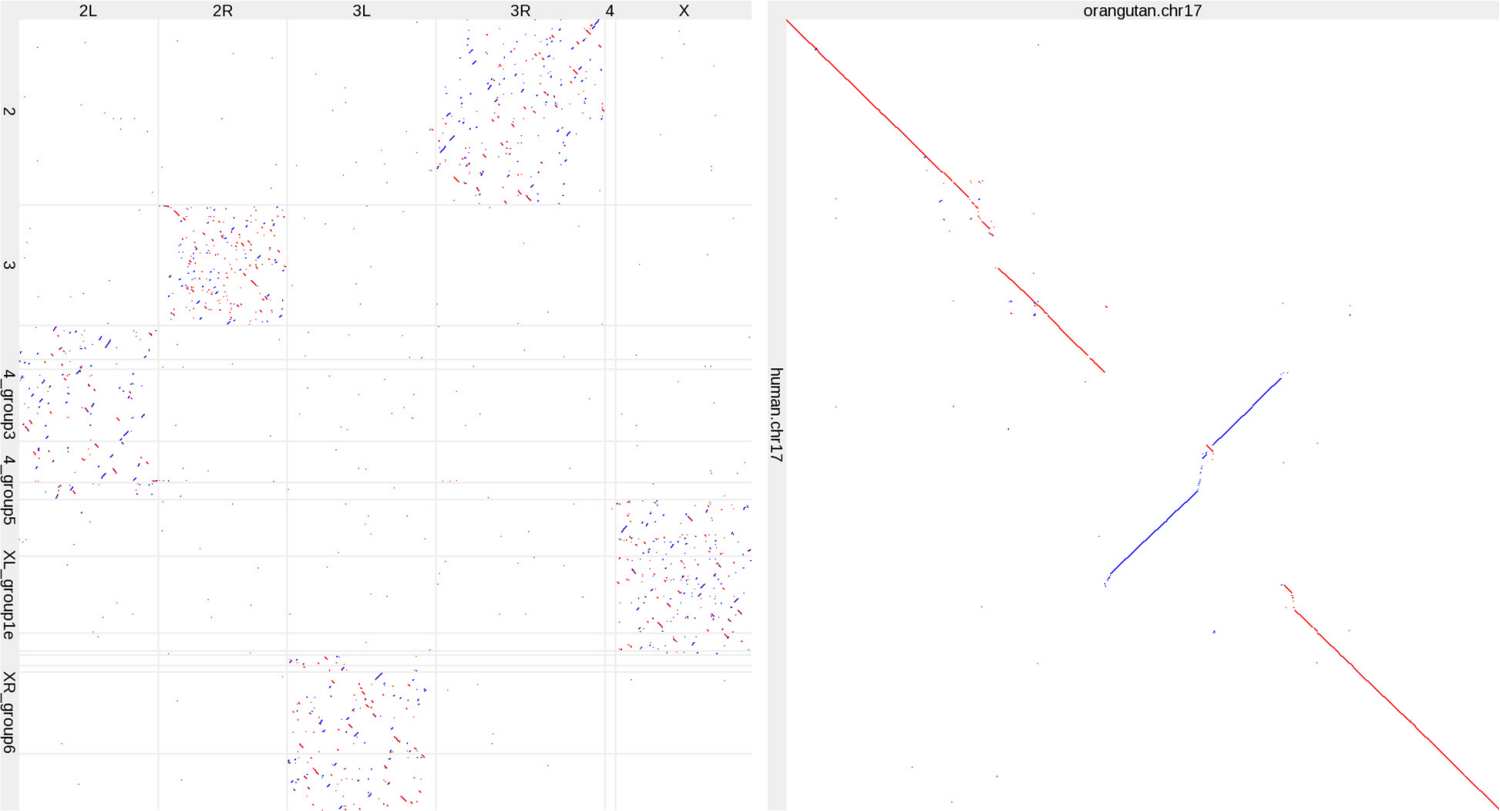
Genome alignments. Left: *D. melanogaster* (horizontal) versus *D. pseudoobscura* (vertical). Right: orangutan versus human chromosome 17. Red indicates same-strand alignments and blue indicates opposite-strand alignments

The original meaning of “syntenic” is “on the same chromosome” [9]. Thus “conserved synteny” means conservation of being on the same chromosome. Comparison of *Drosophila melanogaster* and *Drosophila pseudoobscura* genomes shows striking synteny conservation: although these genomes are highly shuffled relative to each other, the shuffling is mostly within and not between chromosomes (Fig. 2).

Another pattern is conserved order and orientation. This happens when an ancestral genomic segment has been partially rearranged by inversions, deletions, insertions, etc, but parts of it remain in their ancestral order and orientation. This can be seen in human and orangutan chromosomes 17 (Fig. 2). Most genome alignment methods use conserved order and orientation to help construct their alignments [10].

### Alignment methods

The classic approach to alignment is to define a scoring scheme, with substitution and gap scores (e.g. Table 1), and then seek alignments with maximal total score. This is equivalent to using a statistical model of related sequences, with substitution and gap probabilities, and seeking alignments with maximal likelihood under the model [11, 12].

**Table 1:** Alignment scoring schemes used in this study, and their underlying probabilities

human-chimp.v2	HoxD70 [48]	HoxD55			
a c g t	a c g t	a c g t			
a 90 -330 -236 -356	a 91 -114 -31 -123	a 91 -90 -25 -100			
c -330 100 -318 -236	c -114 100 -125 -31	c -90 100 -100 -25			
g -236 -318 100 -330	g -31 -125 100 -114	g -25 -100 100 -90			
t -356 -236 -330 90	t -123 -31 -114 91	t -100 -25 -90 91			
gap existence cost: 600	gap existence cost: 400	gap existence cost: 400			
gap extension cost: 150	gap extension cost: 30	gap extension cost: 30			
a c g t	a c g t	a c g t			
a.27.00052.0020.00041	a.18.019.045.020	a.16.028.050.029			
c.00052.23.00053.0020	c.019.16.015.045	c.028.13.022.050			
g.0020.00053.23.00052	g.045.015.16.019	g.050.022.13.028			
t.00041.0020.00052.27	t.020.045.019.18	t.029.050.028.16			
gap existence probability:.000021	gap existence probability:.043	gap existence probability:.091			
gap extension probability:.11	gap extension probability:.73	gap extension probability:.76			

It is said that “all models are wrong, but some are useful”, and this is no exception. This model lacks many features of related sequences: substitutions are more frequent at CG dinucleotides, indels are more common in tandem repeats, some regions (e.g. protein-coding) are more conserved than others, structural RNA genes conserve complementarity rather than primary sequence, etc. There have been proposals to model some of these features (e.g. [13–15]), but they have a cost in run time and nuisance parameters. In this study we shall just use the classic alignment model, though our new methods could be combined with more complex models. Classic alignment has been very widely used, and often works well enough to give useful results. It can successfully align orthologs whose primary sequence is not constrained, provided their common ancestry is recent enough that they have not diverged too far.

Maximal-score alignment has an under-appreciated flaw: it can spuriously align dissimilar and unrelated sequences, if they are flanked by similar sequences [16]. Although the dissimilar sequences will have negative alignment score, if both flanks have positive scores of greater magnitude then the score is maximized by aligning the whole thing. The underlying problem is that this approach seeks optimal individual alignments, but we really want an optimal set of alignments.

Maximal-score alignments can be found by the Smith-Waterman-Gotoh algorithm [17, 18], but this is slow for large genomes and so fast heuristics are used instead. A typical heuristic is seed-and-extend, which often has three steps: 1) find “seeds”, i.e. short matches that can be found quickly; 2) for each seed check whether there is a gapless alignment with score ≥ some threshold *d*; 3) if so check whether there is a gapped alignment with score ≥*e*.

Step 3 is often done with a “gapped *x*-drop algorithm” [19, 20]. This means that we try extending an alignment in all possible ways, with any pattern of insertions and deletions, but stop if the score drops more than *x* below the maximum seen so far. It can be argued that *x* should be just less than *e*: lower values of *x* can hide alignment flanks with positive score, but higher values cause trouble by merging alignments with score *s*≥*e* across drops with score ≤−*s* [21].

### Repeat masking

Repeats (interspersed repeats and simple sequences) are typically masked before alignment. Specifically, they are marked using lowercase letters, seeds are forbidden from overlapping them, but the final alignments are allowed to extend into them. A major reason for masking is to make the computation tolerable: without it, e.g. each of the 1 million human Alu repeats would hit each of the 1 million chimp Alus, producing 10^12^ alignments.

### Summary of this study

This study presents a new genome alignment method, with several interesting features:

- It is based on finding an optimal set of alignments, instead of optimal individual alignments.
- It aligns without masking, which turns out to be important for orthology search.
- It uses a statistical model to estimate the reliability (unambiguity) of each alignment part, enabling the user to disregard less-reliable parts.
- In a major departure, it does not consider conserved order and orientation. Although considering this is sensible, the ways that other aligners do so are problematic.

Compared to previous aligners, this method aligns thousands of loci differently and with much higher similarity, strongly suggesting that the previous alignments are not orthologous.

## Results

### Idea of the new method

The idea is to seek a set of one-to-one alignments between two genomes that maximizes:

(1) $$ \sum\limits_{\text{alignments}} (\text{alignment score} - f)  $$

Here, *f* is an “alignment existence cost”, which is necessary to avoid trivial solutions with lots of length-1 alignments. It is similar to Mauve’s breakpoint penalty [22].

The one-to-one requirement means that each basepair in either genome must match at most one basepair in the other genome. This is crude but tractable, and the hope is it will mostly find one-to-one orthologs. It is akin to the reciprocal best match approach to protein orthology.

This simple scoring system is a natural way to find a set of items. One property is that no alignment can contain any segment with score <−*f*, because in that case the score could be increased by splitting the alignment into two parts either side of the segment. So it solves the aforementioned problem of arbitrarily bad segments in individual alignments. The constant *f* reflects uniform probabilities, in a statistical model, of starting and ending a new item (see the Appendix).

Note this is not equivalent to finding non-overlapping alignments with score >*f*, with a classic aligner like BLAST or WU-BLAST [23, 24]. Our approach optimizes the set rather than individual alignments: for instance, if two alignments overlap, our approach optimizes the breakpoint for jumping between them.

### Algorithm overview

Unfortunately, there does not seem to be an efficient algorithm to find such an optimal set of alignments. The nearest thing is the “repeated matches” algorithm, which finds an optimal set of many-to-one alignments [11]. This is asymmetric: it aligns each basepair in the “query” genome to at most one basepair in the “reference” genome, but not necessarily vice-versa. It is about as fast as Smith-Waterman-Gotoh. In practice, the new method uses these steps:

1. Find local alignments between the two genomes, by seed-and-extend (many-to-many).
2. Apply the repeated matches algorithm, constrained to the candidate alignments found in step 1. We refer to this constrained version of the repeated matches algorithm as “split-alignment”.
  Split-alignment guarantees to find a set of many-to-one alignments that maximizes the sum of (alignment score−*f*), where each alignment in the set is part (or all) of a candidate alignment. In other words, given a set of alignments that overlap in the query, it finds an optimal set of nonoverlapping alignment parts. One aspect of this is finding optimal breakpoints for jumping between overlapping alignments. The output may include multiple parts of one candidate alignment.
3. Perform split-alignment a second time, after swapping the roles of query and reference. This produces one-to-one alignments.

Step 1 uses LAST (though other aligners could be used), and for brevity let us refer to the whole new method as LAST [25]. We shall refer to the output of step 2 as “1-split” alignments, and the output of step 3 as “2-split” alignments.

Many of the following results use the 1-split alignments, because they are easier to evaluate: if we find many-to-one alignments between genomes Q (query) and R (reference), we can assess whether each alignment could be improved by aligning the same segment of Q to a different region of R. They are also more comparable to the UCSC genome alignments, which are many-to-one [26, 27].

### Statistical model

By using a probabilistic version of split-alignment (a kind of Forward-Backward algorithm [11], see the Appendix), we can estimate the probability that each pair of bases is wrongly aligned. This is high if that region of genome Q aligns almost equally well to other regions of genome R. The following results omit alignments from each set that lack at least one position with error probability ≤ 0.00001.

### Results with pre-masking

**Fig. 3:**
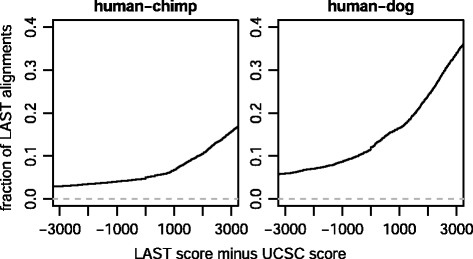
Comparison of LAST (1-split, pre-masked) and UCSC genome alignments. The panel headings show **query-reference**. For each “different” LAST alignment (no pair of aligned bases in common with UCSC) whose human segment is covered by one UCSC alignment, that segment’s alignment scores are compared

The new method was used to align the human and chimp genomes, with standard repeat-masking at first. To facilitate comparison with the UCSC alignments, the same scoring scheme was used (human-chimp.v2, Table 1). This produced 371977 1-split alignments (with human as query), of which 15084 are “different” from UCSC, meaning no pair of aligned bases in common. For 6845 of these different alignments, the alignment’s human segment is 100 % covered by (i.e. contained in) one UCSC alignment: so we can compare the alignment scores for this (exact same) human segment. LAST’s score is higher in 95 % of cases (Fig. 3). For human versus dog, LAST’s score is higher in 90 % of cases.

It is encouraging that LAST usually gets higher scores, but the 5–10 % of lower scores are clear failures in its aim of finding an optimal set of alignments. Inspection of several cases revealed that these failures are caused by masking. If the true ortholog of a sequence is masked, but a paralog is not, then LAST may incorrectly align the paralog. Fundamentally, masking is dangerous for orthology search in a way that it is not for homology search. In homology search it can only cause false-negatives, but in orthology search it can also cause false-positives.

### Alignment without masking

We would thus like to align without masking, but we still wish to avoid aligning independently-evolved simple sequences (Fig. 1a). This was achieved by post-masking: alignments that mostly overlap simple sequence were discarded at the end.

The problem is that alignment without masking takes much longer and produces overwhelming output (Table 2, row “mask” versus row “unmask”). It is feasible because we use LAST, whose seeds adapt (in length and rareness) to repeats [25]. So the number of seed hits merely doubles (because about half the query was previously masked). The main problem is that the number of gapless alignments increases 100-fold. This is because a greater proportion of the seed hits lie in high-scoring alignments (repeats).

**Table 2:** Statistics for aligning human chromosome 22 to the chimp genome (step 1 only: no split-alignment)

Method	Seeds	Alignments ×10^3^		Time	Output
	×10^6^	gapless	gapped	(min)	(MB)
mask	856	1641	33	7	110
unmask	1881	161551	118308	583	72945
cull	1881	26740	12243	93	7479

To mitigate this problem, a “gapless alignment culling” step was added. This step discards any gapless alignment whose query segment lies in those of two or more other alignments with greater score-per-length.^a^ This aims to get the strongest matches to each region of the query (like adaptive seeds), not all matches. Ultimately we just want one strongest match, but the second-strongest helps us to calculate model probabilities. A similar culling procedure is present in BLAST [28].

### Results with post-masking

**Fig. 4:**
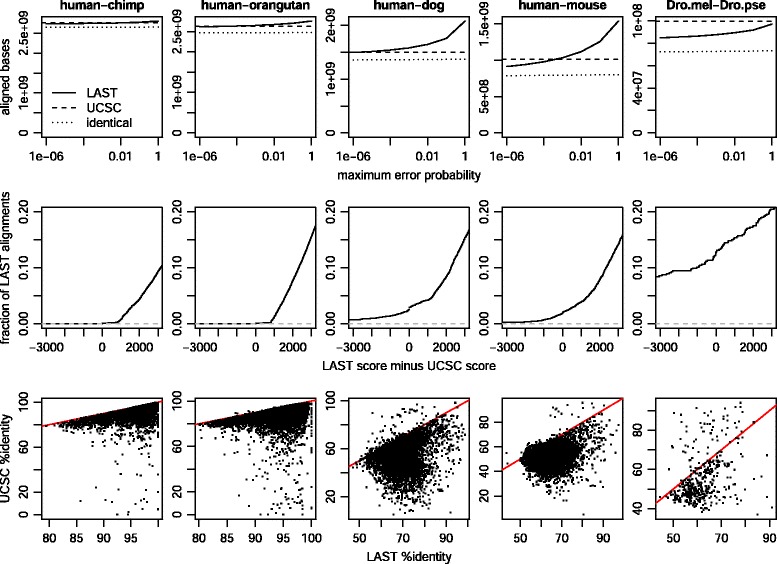
Comparison of LAST (1-split, post-masked) and UCSC genome alignments. The column headings show **query-reference**. Top row: number of aligned bases by LAST, as a function of max error probability, and the number of these aligned identically by UCSC. 2^nd^ row: score difference, for each “different” LAST alignment (no pair of aligned bases in common with UCSC) whose query-genome segment is covered by one UCSC alignment. 3^rd^ row: %identity comparison, for the same alignments as in row 2. Red lines indicate equal %identity

Post-masking (of simple sequences only, not interspersed repeats) was tested on five pairs of genomes (Fig. 4). In each case, the majority of aligned bases are identical to the UCSC alignments (Fig. 4, top row), but a nontrivial proportion are different (Table 3). For example, in the human-mouse comparison, >12 % of aligned bases lie in alignments that are completely different from UCSC (no pair of aligned bases in common).

**Table 3:** Quantities of LAST (1-split, post-masked) alignments, and differences from UCSC alignments

Genomes	Alignments	Different ^*b*^	Moved ^*c*^	New ^*d*^
	(bases ^*a*^)	(bases ^*a*^)	(bases ^*a*^)	(bases ^*a*^)
human-	435084	51208	7591	31184
chimp	(2.7e9)	(6.5e7)	(1.4e7)	(2.9e7)
human-	911016	112221	19050	63481
orang	(2.6e9)	(1.1e8)	(2.0e7)	(5.2e7)
human-	1626114	234267	5203	182648
dog	(1.5e9)	(1.1e8)	(2.2e6)	(9.1e7)
human-	1150523	275161	6763	226204
mouse	(9.4e8)	(1.2e8)	(2.5e6)	(9.7e7)

^*a*^number of query basepairs that are aligned to a reference basepair ^*b*^alignments (bases therein) that have no pair of aligned bases in common with UCSC ^*c*^“different” alignments (bases therein) whose human segment is covered by one UCSC alignment ^*d*^alignments (bases therein) whose human segment is completely unaligned by UCSC

As above, we can compare scores for “different” LAST alignments whose human segment is covered by one UCSC alignment (Fig. 4, row 2). For the ape comparisons, LAST’s score is almost always higher, so post-masking does indeed improve the results. Moreover, the LAST scores are higher by a margin of at least 795: this comes from the error probability threshold of 0.00001, because a score difference of 795 is equivalent to a 10^5^-fold difference in model probability.

The human-dog and human-mouse results are not quite as good: the LAST scores are lower in about 2 % of cases. This is at least partly because these genomes are more diverged, so LAST’s seeds miss some orthologs.

It may be more intuitive to compare the LAST and UCSC alignments by %-identity (Fig. 4, row 3). The LAST alignments almost always have higher %-identity, often by a considerable margin, e.g. 10 % or 20 %. %-identity can be misleading, because it treats e.g. one length-10 gap the same as 10 substitutions. It is better to weight different types of change by their evolutionary likelihoods, which is done in the alignment scores (Fig. 4, row 2).

**Fig. 5:**
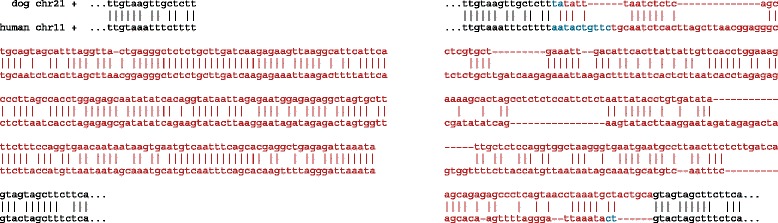
Example of an inversion wrongly aligned in un-inverted orientation by UCSC. Left: LAST alignments, with the inversion in red. Right: UCSC alignment, with the incorrect part in red, and bases unaligned by LAST in blue

In summary, there are thousands of loci that LAST aligns completely differently from UCSC, with significantly higher score and %-identity. Some overlap protein-coding exons (Table 4). It is plausible that in many of these cases the LAST alignments are orthologous and the UCSC alignments are not. In some cases, the UCSC alignments lack similarity and homology. An example is shown in Fig. 5, where UCSC aligns an inversion in un-inverted orientation. In other cases, the UCSC alignments are homologous, but less similar than the LAST alignments. However, UCSC favours chains of colinear alignments, and we may wonder whether we would rather have (say) a 91 %-identity colinear human-chimp alignment or a 98 %-identity non-colinear alignment (Table 4). When the difference in similarity is this large, it is more plausible that the UCSC alignment is paralogous. Since paralogs often come from tandem duplication, they can lie in chains. Several factors may cause lower-similarity chained alignments.

- Large and complex duplications: these create ambiguity about how to construct chains.

**Table 4:** Examples of better human-chimp alignments found by LAST than UCSC (mm=mismatches)

Human segment	LAST alignment			UCSC alignment(s)			Gene
	%id	mm	gaps	%id	mm	gaps	
chr1:152276674–152277614	97	30	0	92	76	3	FLG
chr1:152280487–152281478	97	30	0	92	77	3	FLG
chr2:108873888–108876032	99	29	1	88	220	35	SULT1C3
chr2:132248761–132251678	98	40	23	94	96	97	MZT2A
chr6:26521953–26522872	99	7	0	91	70	11	HCG11
chr6:161039352–161043226	96	119	21	91	293	50	LPA
chr9:140099185–140099970	100	1	0	97	25	0	TMEM203
chr11:67762787–67763389	99	6	1	95	28	3	UNC93B1
chr15:28386144–28386780	98	8	2	91	50	5	HERC2
chr17:36633111–36634556	99	31	1	97	41	5	ARHGAP23
chr17:36634558–36635933	98	16	11	94	62	16	ARHGAP23
chr19:53078564–53079296	97	14	8	77	68	105	ZNF701
chr19:55262747–55265365	97	71	12	94	148	14	KIR2DL3
chr22:16286739–16288612	96	42	27	90	131	56	POTEH
chrX:3228654–3232013	99	30	2	90	230	106	MXRA5
chrX:3558846–3560429	98	21	8	94	77	19	PRKX
chrX:48112039–48118891	98	100	53	92	461	109	SSX1
- Rearrangement (e.g. inversion) of the ortholog but not the paralog.
- Genome misassembly: most of these assemblies are unfinished drafts. Misassembly is especially likely in regions with complex duplications, repeats, and rearrangements.
- Gene conversion: this can convert an ortholog to a paralog.
- Contaminating human sequence in e.g. the chimp assembly.
- Accelerated evolution: this can decrease the similarity of an ortholog.

### Wrong *x*-drop alignments

**Fig. 6:**
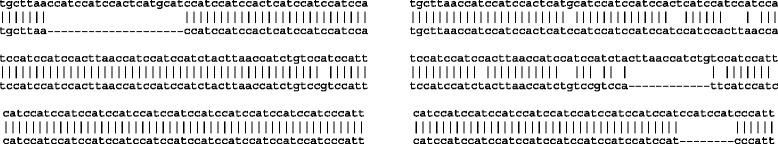
Example of wrong alignment by the gapped *x*-drop heuristic. Left: correct alignment, with a score drop >*x*. Right: incorrect alignment of the same sequences

LAST’s ape comparisons still have a tiny fraction of alignments with lower score than UCSC. These are mostly caused by a pathology of the gapped *x*-drop heuristic (Fig. 6). If an alignment has a region with score <−*x* (e.g. a large gap), the left and right flanks of that region will usually be found as separate alignments. Unfortunately, it is sometimes possible to find an alternative, wrong alignment of the whole region without a score drop >*x*, but with lower score overall. If orthologs are wrongly aligned in this way, the alignment score may be lower than that of paralogs, causing LAST to prefer a paralogous alignment.

This problem can be fixed by either increasing *x* so that the correct alignment is found, or decreasing *x* so that the incorrect alignment is not found and the correct alignment is found in two parts. Unfortunately, different values of *x* fix different cases, and there is no reasonable value that fixes all cases.

### New alignments of repeats

**Fig. 7:**
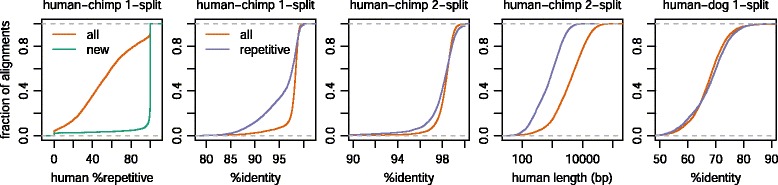
Properties of new and repetitive LAST alignments. “New” means LAST alignments whose human segment is completely unaligned in UCSC. “Repetitive” means LAST alignments whose human segment is 100 % covered by repeat element(s)

The LAST alignments include many cases where the human segment is completely unaligned by UCSC (Table 3). These alignments tend to be covered by repeat elements, such as LINEs and SINEs (Fig. 7, left panel). Many repeats can be aligned unambiguously because they are older than the common ancestor of the genomes, so they have unique orthologs with higher similarity than the other copies. In addition, there are many cases where repeat elements have been inserted within other repeats, creating unique mosaics. Alignment without masking reveals many such potentially interesting orthologies.

Nevertheless, orthology search is likely harder for repeats than non-repeats. The human-chimp 1-split alignments mostly have around 98 % identity, but many of the repeat alignments have lower %-identity (Fig. 7, panel 2). The likely explanation is that many of these repeat alignments are paralogous.

This problem mostly vanishes in the final 2-split alignments (Fig. 7, panel 3). Now the repeat alignments also have around 98 % identity, although they have slightly higher variance: they more often have both higher and lower %-identity. This higher variance is not surprising as repeat alignments tend to be shorter (Fig. 7, panel 4), simply because longer alignments are less likely to be 100 % repetitive.

Surprisingly, the human-dog 1-split repetitive alignments do not have reduced %-identity (Fig. 7, panel 5). A possible explanation is that the human-chimp paralogous alignments are mostly due to poor genome assembly: orthologous human-chimp repeats are often very young, with low divergence, and thus hard to assemble.

### Badness of HoxD55

Alignment of the *D. melanogaster* and *D. pseudoobscura* genomes worked less well: the LAST scores were lower than the UCSC scores in 13 % of cases (Fig. 4). This was the only comparison to use the HoxD55 scheme (Table 1). Inspection of several cases revealed that the LAST failures are due to the *x*-drop problem described above, which evidently occurs much more often with HoxD55. This scoring scheme has a high tolerance for aligning unrelated sequences [29], which presumably exacerbates the *x*-drop error.

**Fig. 8:**
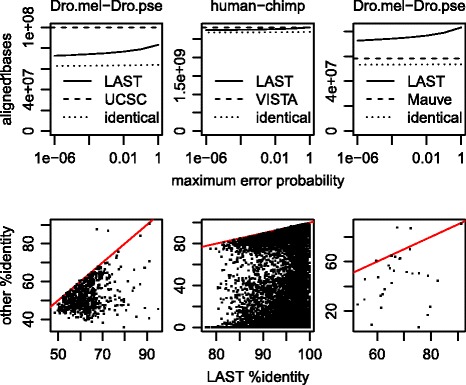
Comparison of LAST (1-split, post-masked) and other genome alignments. Top row: number of bases aligned by LAST, as a function of max error probability, and the number of these aligned identically by the other method. 2^nd^ row: %-identity comparison, for each “different” LAST alignment (no pair of aligned bases in common with the other method) whose query-genome segment is covered by one other-method alignment

Accordingly, the alignment worked much better with HoxD70 (Fig. 8, left column). Now, the %-identity is almost always higher for LAST than UCSC, apart from just two clearly-wrong LAST alignments, caused by *x*-drop error.

### Comparison to other aligners

Many genome alignment methods have been proposed, though most have in common an approach of looking for chains of colinear alignments. In addition to UCSC, let us consider VISTA [30] and Mauve [22] as representative examples.

In the VISTA human-chimp alignments, the vast majority of aligned bases are identical to LAST (Fig. 8). Nevertheless, there are many LAST alignments that have no aligned bases in common with VISTA: for some of these, the human segment is covered by one VISTA alignment, in which case we can compare the %-identities for that human segment. The VISTA %-identity is almost always lower, often much lower (Fig. 8). In fact, VISTA has many more very low %-identity alignments (e.g. <60 *%*) than UCSC (Fig. 4, lower-left panel). Inspection of several cases revealed errors similar to that in Fig. 5. The likely reason is that VISTA uses colinearity more aggressively than UCSC, by globally aligning genome regions defined by chains.

As another comparison, the two Drosophila genomes were aligned using progressiveMauve version 2.3.1 with default parameters. The result is conservative, with fewer aligned bases than LAST, and few cases where Mauve aligns the same region of *melanogaster* to a different region of *pseudoobscura* (Fig. 8). In these few cases, Mauve’s %-identity is usually much lower, apart from the same two LAST errors mentioned above. Although Mauve also uses aggressive global alignment, it subsequently detects and removes alignments of unrelated sequences, to avoid errors like that in Fig. 5 [22, 31].

### Score/model parameters

Good alignment depends on using reasonable score/model parameters (Table 1), and we can check whether they match the substitution and gap frequencies in the alignments. This is only a rough check, because the alignments are not perfect: in particular, the gap existence counts may be underestimates due to “gap attraction” and “gap annihilation” [13, 32].

The main observation is that the gap costs for human-chimp.v2 are unduly large: a better fit would be obtained with a gap existence cost of 500 and a gap extension cost of 30. So we re-did the ape alignments with these costs, then re-counted substitutions and gaps.

**Fig. 9:**
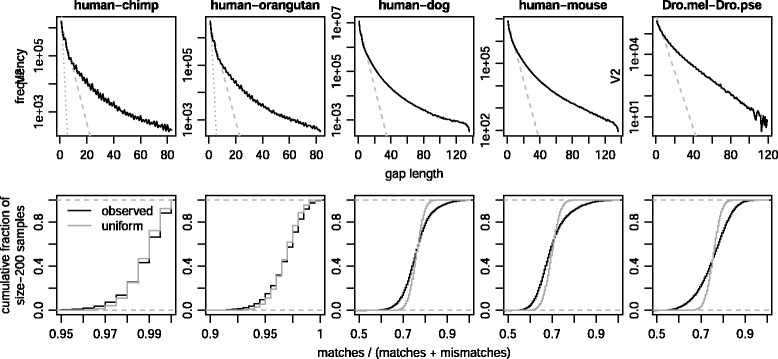
Deviations from the alignment model in LAST (2-split, post-masked) genome alignments. Upper row: gap length distributions. Dotted lines show the distribution modeled by a gap extension cost of 150, and dashed lines a cost of 30. Lower row: substitution rates in 200bp windows (excluding gaps). Grey lines show expected results for uniform substitution rate

The next observation is that gap lengths do not fit any model with a simple gap extension probability, because the frequencies of longer gaps decrease more slowly (Fig. 9). A pragmatic solution is to fit the gap extension probability to short gaps.

The substitution and gap frequencies, and corresponding scores, are shown in Appendix C. They do not differ greatly from the alignment models. However, these frequencies are not uniform across the genome, and averaged parameters may not be ideal. For example, a (match score):(mismatch cost) ratio of 1:1 is appropriate for ∼75 % identity, 1:2 for ∼95 % identity, and 1:3 for ∼99 % identity [33]. To investigate, we deleted gap columns then measured substitution rates in 200bp windows of the alignments (Fig. 9, row 2). The substitution rates are not uniform, but they do not vary arbitrarily: for instance, human-mouse alignments hardly ever have ≥90 % identity. In summary, the used parameters are not wildly unreasonable.

### Alignathon simulation test

Finally, we tested our method on the “Alignathon” simulated genomes [34]. Simulation has the advantage that the true alignments are known, but the disadvantage that the simulation’s realism is unknown. For instance, the simulation presumably lacks rearrangements like that in Fig. 1d.

There are two simulations: one of four ape-like genomes, and one of five mammal-like genomes. The “truths” are multiple (not pair-wise) alignments, and, in our understanding, they align all homologs (including paralogs, but excluding mobile element insertions) that have duplicated since the most recent common ancestor of the genomes. This unfortunately does not match our approach of finding one-to-one orthologs. In any case, for each simulation we made pair-wise alignments with LAST, then joined them into multiple alignments with mafTransitiveClosure [34], which joins pair-wise into multiple alignments in a naïve way.

For the ape simulation, LAST achieved a precision of 0.998 and a recall of 0.978. All other aligners had lower precision (Table S13 in [34]). For the mammal simulation, LAST achieved precision=0.827 and recall=0.612. Several aligners have higher precision, however all but one of those have much lower recall (Tables S15–16 in [34]). The exception is Cactus, with precision=0.885 and recall=0.734.

To understand why Cactus has higher precision, let us focus on pair-wise alignments between simHuman and simMouse. LAST aligns 124 million pairs of bases, of which 25 million are wrong, and 464 thousand lie in completely-wrong alignments (no pair of aligned bases in common with the truth). Cactus aligns 131 million pairs of bases, of which 18 million are wrong, and 6 million lie in completely-wrong alignments. So LAST is much better at avoiding completely-wrong alignments, whereas Cactus excels at accuracy of partly-right alignments. The latter is not surprising, because Cactus is a true multiple aligner: it takes pair-wise alignments from an external source (potentially LAST), and combines and refines them by integrating the information from all the sequences [35].

## Discussion

The new genome alignment method is conceptually extremely simple, it just seeks an optimal set of one-to-one alignments. Despite decades of extensive research on alignment, alignment *sets* have been surprisingly neglected, although they are often what is really wanted, e.g. for multi-domain proteins.

The new method is obviously crude, because it ignores phylogeny and many-to-many orthology. It will fail in cases of reciprocal gene loss, where one copy of a paralog is absent in one genome and the other copy is missing from the other genome. Such hidden paralogy is a major problem in understanding evolution [36].

Nevertheless, the new method seems to fix thousands of non-orthologous parts in previous genome alignments. The previous errors were caused by an over-aggressive assumption of conserved order and orientation. For example, in many cases in Table 4, UCSC finds the same alignment as LAST in its initial (many-to-many) “chains” but omits it from its final (many-to-one) “net” alignments, because it prefers weaker alignments in stronger chains. There is a widespread paradigm of trying to align long colinear blocks (often using “chains” or “anchors”), which risks producing non-orthologous or even non-homologous alignments. The ideal approach is probably to use a weaker preference for conserved order and orientation, e.g. via prior probabilities in a statistical model.

The use of a probabilistic model is a key advantage, since it quantifies the ambiguity of each aligned base. Similar probabilistic methods have been applied before to individual alignments [11, 13, 14, 21], but apparently not to alignment sets.

We found that pre-masking is dangerous for orthology search, which is probably not widely recognized since it is not dangerous for typical BLAST homology searches. Unfortunately, genome alignment without masking is much more compute-intensive, even with adaptive seeds and gapless alignment culling. Probably, better heuristics could be developed to tackle this.

We also found that the gapped *x*-drop heuristic can sometimes produce bad alignments (Fig. 6). This is important because *x*-drop is widely used (e.g. BLAST), the bad alignments are not immediately obvious (probably they are usually overlooked), and this problem does not seem to have been described before. Unfortunately, it is unclear how to fix it, save by applying the repeated matches algorithm directly to the genomes (which seems feasible on a large supercomputer).

Split-alignment has applications beyond whole genome comparison. It can be used to map DNA or RNA reads to a genome. “Mapping” is orthology search (since paralogs are not wanted), and reads are genome fragments (possibly rearranged), so it is all the same thing. Since different reads may redundantly cover the same query bases, we would seek many-to-one alignments, i.e. stop at the 1-split stage. Our method incorporates fastq quality data into the model and scoring [37]. The statistical model, which quantifies the (un)ambiguity of each alignment part, is a major benefit for finding reliable rearrangement breakpoints.

## Conclusions

The new method aligns the majority of genomic bases identically to previous methods, as expected. Nevertheless, around 100 million human bases, which overlap a number of protein-coding regions, are in completely different alignments. The new alignments should be especially beneficial when searching for interesting and unusual features in genome evolution, because these are particularly confounded by alignment errors. One example is accelerated evolution, which is mimicked by paralogy. Another is micro-rearrangements, which are systematically missed in standard genome alignments based on colinearity [38, 39]. Indeed the new alignments suggest many interesting rearrangements (e.g. Fig. 5), but unfortunately it is not straightforward to tell true rearrangements from assembly errors. The new alignments are available at: [40]. The software is available at [41], and also in the last-align package for Debian and Ubuntu [42].

## Materials and methods

### Split-alignment algorithm

**Fig. 10:**
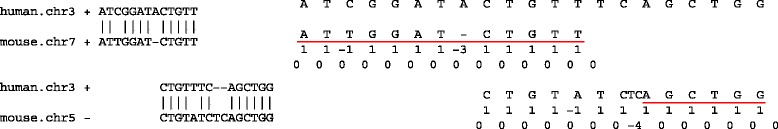
The split-alignment algorithm. Left: input to the algorithm, two local alignments that overlap in the query (top, human) sequence. Right: algorithm layout. This example uses match score=1, mismatch cost=1, and gap cost=2+1 ×gap length. Match, mismatch, and insertion scores (*A*_*ij*_) are written beneath each letter, whereas deletion scores (*D*_*ij*_) are written between letters. The red lines show the optimal split-alignment

The input is a set of local alignments between one query sequence and one genome. (If there is more than one query, the algorithm is applied to each independently.) An example is shown in Fig. 10. First, the alignments are oriented to use the forward strand of the query. *A*_*ij*_ is defined to be the score at query letter *j* in alignment *i*, for match, mismatch, or insertion of this letter. *D*_*ij*_ is defined to be the score between query letters *j*−1 and *j* in alignment *i*, for deletions. The optimal split-alignment score is calculated by dynamic programming, using these recurrence relations:

(2) $$\begin{array}{*{20}l} V_{i\,j+1} = \max(V_{i\,j} + D_{i\,j},\ W_{j} - f) + A_{i\,j} \end{array} $$

(3) $$\begin{array}{*{20}l} W_{j+1} = \max(W_{j},\ \max_{i} V_{i\,j+1}) \end{array} $$

The recurrence is initialized like this:

(4) $$\begin{array}{*{20}l} V_{i\:\text{beg}(i)} = -\infty \end{array} $$

(5) $$\begin{array}{*{20}l} W_{\text{beg}} = 0 \end{array} $$

where beg(*i*) is the coordinate of the first query letter in alignment *i*, and beg= min(beg(*i*)). The optimal split-alignment score is *W*_end_, where end= max(end(*i*)), and end(*i*) is one-past the last query letter in alignment *i*. This only calculates the score, but an optimal split-alignment can then be found by a standard traceback procedure [11].

### Genome data

These assemblies were used: panTro4, ponAbe2, canFam3, mm10, dp4, dm3 (without chrUextra), and hg19 (without alternate haplotypes and with the chrY pseudo-autosomal regions replaced by ‘n’s).

The UCSC genome alignments were taken from the axtNet subdirectories of these directories: hg19/vsPanTro4, hg19/vsPonAbe2, hg19/vsCanFam3, hg19/vsMm10, dm3/vsDp4.

The VISTA alignment was taken from: [43].

### Pre-masking

Lowercase-masked genomes were obtained from the UCSC database. Tandem repeats found by tantan version 13 were additionally masked, in order to prevent non-homologous alignments more reliably [1].

### Post-masking

The genomes were lowercase-masked by tantan only, then aligned case-insensitively, and at the very end each alignment was rescored with gentle masking of lowercase letters [2]: if it lacked any segment with score ≥*e* it was discarded.

### Seed patterns

The sensitive transition seed set MAM8 was used by default [44]. For the closely-related apes, the spaced seed 1111110 was used instead. For human-dog and human-mouse with post-masking, since the number of indexed bases roughly doubles without masking, MAM4 was used to avoid a too-large index.

### Alignment parameters

LAST’s seed rareness limit *m* was empirically set to 50 for the ape alignments and 100 for the others. The score threshold *e* was set to values with borderline statistical significance, using ALP [45]: 5000 for the flies with HoxD55, 4000 for the flies with HoxD70, 4500 for mammals with HoxD70, and 3000 for the apes. The alignment existence cost *f* and the maximum gapped score drop *x* were both set to *e*−1.

### Alignment commands

To illustrate, the Drosophila HoxD70 alignments can be constructed with LAST v535 as follows. First, run tantan on both genomes, with default settings. Then, make the 1-split alignments like this: lastdb¡-uMAM8¡x¡dp4.fa lastal¡-pHOXD70¡-e4000¡-C2¡-m100¡x¡dm3.fa¡| last-split¡-m1¡>¡1.maf Next, make the 2-split alignments like this: maf-swap¡1.maf¡|¡last-split¡-m1¡>¡2.maf Finally, run last-postmask on 1.maf and 2.maf.

### Alignathon ape test

These query-reference pairs were aligned: simChimp-simHuman, simGorilla-simHuman, simOrang-simHuman. The alignment procedure was the same as for the real ape genomes (lastdb option -m1111110, and lastal options -phuman-chimp.v2 -a500 -b30 -e3000 -C2 -m50). Alignments with error probability ≤ 0.00001 were retained, and joined by mafTransitiveClosure.

### Alignathon mammal test

These query-reference pairs were aligned: simDog-simHuman, simMouse-simHuman, simRat-simMouse, simCow-simDog. Since the simulated genomes are smaller than the real ones, we used MAM8 instead of MAM4 (lastdb option -uMAM8, and lastal options -pHOXD70 -e4500 -C2 -m100). Alignments with error probability ≤ 0.00001 were retained, and joined by mafTransitiveClosure.

### Data availability

The data set supporting the results of this article is available in the Zenodo repository [46].

## Endnote

^a^ Score-per-length is computed for whole alignments, not overlapping parts.

## Appendix A: Statistical models

The aim here is to explain and motivate the statistical model of alignments, and the *f* parameter (alignment existence cost). It is instructive to first consider models of segments, such as hydrophobic segments in protein sequences. Segments are a simpler (1-dimensional) analog of alignments.

### A.1 Segments

A simple model is for segments to have independent letters with frequencies *π*_*x*_, while background (non-segment) regions have letter frequencies *θ*_*x*_. Given a sequence, we can then seek maximal-likelihood segments.

**Fig. 11:**
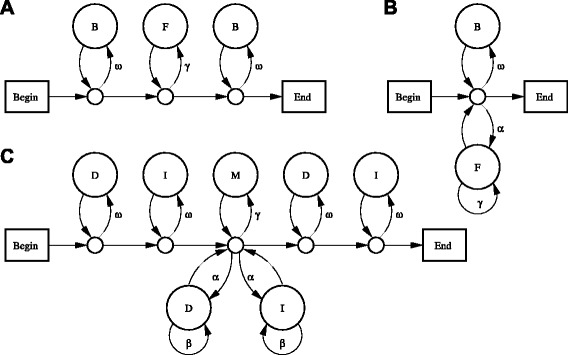
Probabilistic models for segments and local alignments. **a** Segment model. **b** Segment set model. **c** Alignment model. States labeled B (background) emit letter *x* with probability *θ*_*x*_. States labeled F (foreground) emit letter *x* with probability *π*_*x*_. The state labeled M (match) emits aligned letters *x*:*y* with probability *π*_*xy*_. States labeled D (delete) emit reference letters *x* with probability *ϕ*_*x*_. States labeled I (insert) emit query letters *y* with probability *ψ*_*y*_. Small circles are just connectors and do not emit

Figure 11a shows a precise model of this kind, with transition probabilities *ω* and *γ*, in a standard circle-and-arrow notation [11]. Suppose we have a sequence *Q* of length *n*. Let us calculate the likelihood of the path through the model whereby *Q*_*c*+1_…*Q*_*d*_ is a foreground segment:

(6) $$ {\fontsize{8.8pt}{9.6pt}\selectfont{\begin{aligned} {}\text{prob}(\text{path}, Q) &= \left(\prod\limits_{k=1}^{c} \omega \theta_{Q_{k}} \right) (1-\omega)\\ &\times\left(\prod\limits_{k=c+1}^{d} \gamma \pi_{Q_{k}} \right) (1-\gamma) \left(\prod\limits_{k=d+1}^{n} \omega \theta_{Q_{k}} \right) (1-\omega) \end{aligned}}}  $$

This can be simplified by factoring out a constant *μ*, defined as:

(7) $$ \mu = \left(\prod\limits_{k=1}^{n} \omega \theta_{Q_{k}} \right) (1-\omega)^{2} (1-\gamma)  $$

Because *μ* does not depend on the path, we can find a most-probable path by maximizing:

(8) $$ \frac{\text{prob}(\text{path}, Q)}{\mu} = \prod\limits_{k=c+1}^{d} \frac{\gamma}{\omega} \frac{\pi_{Q_{k}}}{\theta_{Q_{k}}}  $$

Next, because maximizing a value is equivalent to maximizing its logarithm, we can maximize:

(9) $$ \ln \left(\frac{\text{prob}(\text{path}, Q)}{\mu} \right) = \sum\limits_{k=c+1}^{d} \ln \left(\frac{\gamma}{\omega} \frac{\pi_{Q_{k}}}{\theta_{Q_{k}}} \right)  $$

We can now define a scoring scheme, where each letter-type *x* receives a score:

(10) $$ S(x) = t \ln \left(\frac{\gamma}{\omega} \frac{\pi_{x}}{\theta_{x}} \right)  $$

Here, *t* is an arbitrary scale factor. Maximal-likelihood segments are runs of letters with maximal total score. Scores are related to model probabilities like this:

(11) $$ \text{prob}(\text{segment}) \propto \exp(\text{score}(\text{segment}) / t)  $$

### A.2 Segment sets

Figure 11a clearly models *one* segment, and we can instead model multiple segments using Fig. 11b, with transition probabilities *ω*, *γ*, and *α*. It can be shown that a maximal-likelihood segment set is one that maximizes:

(12) $$ \sum\limits_{\text{segments}} (\text{segment score} - f)  $$

Here, the segment score is the sum of the letter scores *S*(*x*), and *f* is:

(13) $$ f = -t \ln (\alpha (1-\gamma) / \gamma)  $$

Thus, a segment existence cost *f* arises naturally from model probabilities of starting and ending a segment.

### A.3 Alignments

Figure 11c shows one possible model of local alignments. It can be shown that a maximal-likelihood alignment is one with maximal score according to this scheme:

(14) $$\begin{array}{*{20}l} S(x, y) = t \ln \left(\frac{\pi_{xy}}{\phi_{x} \psi_{y}} \cdot \frac{\gamma}{\omega^{2}} \right) \end{array} $$

(15) $$\begin{array}{*{20}l} \text{gap existence cost} = -t \ln (\alpha (1-\beta) / \beta) \end{array} $$

(16) $$\begin{array}{*{20}l} \text{gap extension cost} = -t \ln (\beta / \omega) \end{array} $$

In this study, it was assumed that *γ*≈*ω*^2^, and *t* was calculated from each score matrix (Table 5) using the method of Yu et al. [12].

**Table 5:** Score matrix scale factor *t*

Score matrix	*t*
human-chimp.v2	69.0042
hoxd70	96.1735
hoxd55	111.906

### A.4 Alignment sets

Unfortunately, it is unclear how to make a simple model like those in Fig. 11 for a set of local alignments. So let us proceed by brute force. In all three previous models, it was the case that prob∝ exp(score/*t*). We can *define* the probability of any alignment set *A* as follows:

(17) $$ \text{prob}(A) \propto \exp(\text{score}(A) / t)  $$

where

(18) $$ \text{score}(A) = \sum\limits_{\text{alignments}} (\text{alignment score} - f)  $$

The score parameters and *t* are the same as in the single-alignment model, so the only new parameter is *f*.

## Appendix B: Probability calculation

The probabilistic version of the split-alignment algorithm is described here. These exponentiated scores are used:

(19) $$\begin{array}{*{20}l} A'_{i\,j}& =& e^{A_{i\,j} / t} \end{array} $$

(20) $$\begin{array}{*{20}l} D'_{i\,j} & =& e^{D_{i\,j} / t} \end{array} $$

(21) $$\begin{array}{*{20}l} f' &=& e^{f / t} \end{array} $$

The Forward algorithm is:

(22) $$\begin{array}{*{20}l} F_{i\:\text{beg}(i)} = 0 \end{array} $$

(23) $$\begin{array}{*{20}l} G_{\text{beg}} = 1 \end{array} $$

(24) $$\begin{array}{*{20}l} F_{i\,j+1} = (F_{i\,j} D'_{i\,j} + G_{j} / f') A'_{i\,j} \end{array} $$

(25) $$\begin{array}{*{20}l} G_{j+1} = G_{j} + \sum\limits_{i} F_{i\,j+1} \end{array} $$

The Backward algorithm is:

(26) $$\begin{array}{*{20}l} B_{i\:\text{end}(i)} = 0 \end{array} $$

(27) $$\begin{array}{*{20}l} C_{\text{end}} = 1 \end{array} $$

(28) $$\begin{array}{*{20}l} B_{i\,j-1} = (B_{i\,j} D'_{i\,j} + C_{j}) A'_{i\,j-1} \end{array} $$

(29) $$\begin{array}{*{20}l} C_{j-1} = C_{j} + \sum\limits_{i} B_{i\,j-1} / f' \end{array} $$

These algorithms enable us to calculate the model probability of each column in each alignment. The probability for a column in alignment *i* with query letter *j* is:

(30) $$ P_{i\,j} = (F_{i\,j+1} B_{i\,j} / A'_{i\,j}) / z  $$

where *z*=*G*_end_=*C*_beg_. The probability for a column in alignment *i* between query letters *j*−1 and *j* is:

(31) $$ P^{\text{del}}_{i\,j} = F_{i\,j} B_{i\,j} D'_{i\,j} / z  $$

Each column’s error probability is one minus its model probability.

The practical implementation of this Forward-Backward algorithm uses scaling to avoid numerical instability [11].

## Appendix C: Substitution/gap counts

The substitution and gap frequencies in each genome alignment are shown in Table 6. The gap extension probabilities were manually set to the stated values, based on Fig. 9.

**Table 6:** Substitution and gap probabilities and scores inferred from genome alignments

Genomes	Probabilities	*t*	Scores		
	a c g t		a c g t		
	a.29.00053.0022.00044		a 77 -300 -212 -337		
human-chimp	c.00053.2.00054.0022	63.495	c -300 100 -275 -212		
	g.0022.00054.2.00053		g -212 -275 100 -300		
	t.00044.0022.00053.29		t -337 -212 -300 77		
	gap existence probability: 0.00077		gap existence cost: 495		
	gap extension probability: 0.65		gap extension cost: 27		
	a c g t		a c g t		
	a.29.0013.0055.001		a 77 -248 -154 -287		
human-orangutan	c.0013.2.0013.0055	64.4704	c -248 100 -222 -154		
	g.0055.0013.2.0013		g -154 -222 100 -248		
	t.001.0055.0013.29		t -287 -154 -248 77		
	gap existence probability: 0.0018		gap existence cost: 448		
	gap extension probability: 0.65		gap extension cost: 28		
	a c g t		a c g t		
	a.24.012.037.013		a 77 -126 -38 -154		
human-dog	c.012.14.0087.037	79.0646	c -126 100 -121 -38		
	g.037.0087.14.012		g -38 -121 100 -126		
	t.013.037.012.24		t -154 -38 -126 77		
	gap existence probability: 0.012		gap existence cost: 429		
	gap extension probability: 0.73		gap extension cost: 25		
	a c g t		a c g t		
	a.22.016.044.018		a 79 -114 -27 -136		
human-mouse	c.016.13.011.044	86.9541	c -114 100 -115 -27		
	g.044.011.13.016		g -27 -115 100 -114		
	t.018.044.016.22		t -136 -27 -114 79		
	gap existence probability: 0.015		gap existence cost: 451		
	gap extension probability: 0.73		gap extension cost: 27		
	a c g t		a c g t		
	a.21.015. 031.014		a 92 -123 -59 -139		
Dro.mel-Dro.pse	c.015.17.014.031	86.2603	c -123 100 -117 -59		
	g.031.014.17.015		g -59 -117 100 -123		
	t.014.031.015.21		t -139 -59 -123 92		
	gap existence probability: 0.016		gap existence cost: 445		
	gap extension probability: 0.73		gap extension cost: 27		
